# *In vivo* and *in vitro* anti-inflammatory activity of *Cryptostegia grandiflora* Roxb. ex R. Br. leaves

**DOI:** 10.1186/0717-6287-47-32

**Published:** 2014-07-10

**Authors:** Jenny P Castro, Yanet C Ocampo, Luis A Franco

**Affiliations:** Biological Evaluation of Promissory Substances Group, Faculty of Pharmaceutical Sciences, University of Cartagena, Cartagena, 130015 Colombia

**Keywords:** Ear edema, Free radicals, Inflammation, MPO, PGE2, RAW 264.7 macrophages

## Abstract

**Background:**

Despite *Cryptostegia grandiflora* Roxb. ex R. Br. (Apocynaceae) leaves are widely used in folk Caribbean Colombian medicine for their anti-inflammatory effects, there are no studies that support this traditional use. Therefore, this work aimed to evaluate the effect of the total extract and primary fractions obtained from *Cryptostegia grandiflora* leaves, using *in vivo* and *in vitro* models of inflammation, and further get new insights on the mechanisms involved in this activity.

**Results:**

Ethanolic extract of *Cryptostegia grandiflora* leaves, and its corresponding ether and dichloromethane fractions, significantly reduced inflammation and myeloperoxidase activity (MPO) in ear tissue of mice treated with 12-*O*-tetradecanoyl-phorbol-13-acetate (TPA). Histological analysis revealed a reduction of edema and leukocyte infiltration. Complementarily, we demonstrated that extract and fractions reduced nitric oxide (NO•) and prostaglandin E2 (PGE2) production in LPS-stimulated RAW 264.7 macrophages, as well as scavenging activity on DPPH and ABTS radicals.

**Conclusions:**

Our results demonstrated for the first time the anti-inflammatory activity of *Cryptostegia grandiflora* leaves, supporting its traditional use. This activity was related to inhibition of MPO activity, and PGE2 and NO• production. These mechanisms and its antioxidant activity could contribute, at least in part, to the anti-inflammatory effect showed by this plant.

## Background

Harmful stimuli to body, such as pathogens, damaged cells or physical injury, are able to trigger an inflammatory response. When this response is persistent, chronic inflammation appears as an undesirable phenomenon that can lead to the development of severe diseases such as osteoarthritis, rheumatoid arthritis, gout, asthma, inflammatory bowel disease, diabetes, cancer, cardiovascular disease and neurodegenerative disorders, which represent an important cause of morbidity and mortality worldwide [1–3].

Macrophages are considered the main immune effector cells, playing a pivotal role in tissue damage in a great number of inflammatory conditions, both acute and chronic. Activated macrophages are able to synthesize reactive oxygen species (radical superoxide •O_2_^−^, hydrogen peroxide H_2_O_2_, and the highly reactive hydroxyl radical •OH), NO•, arachidonic acid metabolites, and lysosomal enzymes which are necessary to perform their phagocytic function. These cells also play an important role in the development of various chronic diseases such as cancer and allergies [4–6].

Traditional medicine has been a revealing source of new molecules in modern drug discovery, thus the use of herbal medicines or their active components represents an increasingly explored and promissory alternative for the treatment of numerous diseases, including inflammatory disorders [7]. The application of natural products in traditional medical systems of many countries is due to a current trend towards the use of green products because they are considered safer. However, limited scientific evidence regarding to the effectiveness of natural bioactive derivatives, in addition to a limited understanding of the mechanisms of action related with their biological activity, has limited its incorporation into clinical practice [8–10].

Considering the folk use of *Cryptostegia grandiflora* Roxb. ex R. Br. (Apocynaceae) on inflammatory conditions, which was revealed by ethnobotanical studies conducted by research groups at the University of Cartagena in the Colombian Caribbean region, and that no information is available about its topical anti-inflammatory properties, we investigated the effect of the total ethanolic extract and primary fractions obtained from the leaves of this plant using a murine model of topical inflammation. Additionally to further get new insights into the possible mechanisms involved in this activity, we evaluated the effect on PGE2 and NO• production, MPO activity and scavenging of the free radicals DPPH, ABTS, and NO•.

## Results and discussion

### Anti-inflammatory activity

*Cryptostegia grandiflora* also known in Colombian Caribbean coast as “20 de Julio or Flor de Muerto” and rubber vine in English speaking countries, is a perennial woody shrub widely distributed in Madagascar, India, South Florida and tropical regions [11–13]. Due to its high latex content, *Cryptostegia grandiflora* is employed in the manufacture of rubber and as a source of hydrocarbon fuels [11]. However, the interest in this plant is not restricted to its industrial applications, but rather to its usages in folk medicine as hypoglycemic, coagulant, antibacterial, anti-inflammatory, anti-asthmatic and for the treatment of nervous disorders [13–17], representing a promissory source of bioactive secondary metabolites. Consequently, several research groups have corroborated many of the biological activities mentioned above. However, to our knowledge, this is the first report of the anti-inflammatory activity of *Cryptostegia grandiflora* leaves.

TPA-induced ear edema model is a valid model to effectively screen compounds with anti-inflammatory potential. Topical application of TPA induces an inflammatory response associated with edema formation and increased MPO activity [18–20]. This is consistent with the results obtained in our study, where topical application of TPA induced a severe inflammatory response in mouse ear that resulted in massive edema formation which was evident by the increased ear weight in comparison with control group, only treated with acetone (p < 0.001). Total extract and ether and dichloromethane fractions from *Cryptostegia grandiflora* significantly reduced inflammation induced by TPA, with inhibition percentages higher than 40%, similar to that produced by indomethacin, which was used as positive control, whereas methanol fraction did not exert anti-inflammatory effects (Table 1). None of the tested extract/fractions produced evident adverse local or systemic effects, suggesting that their topical administration is well tolerated. Due to the lack of bioactivity of methanol fraction in the *in vivo* model, we selected the total extract and ether and dichloromethane fractions of *Cryptostegia grandiflora* to further evaluate their histopathological effect and the inhibition of MPO activity.

**Table 1:** **Topical anti-inflammatory effect of the total extract and primary fractions obtained from** ***Cryptostegia grandiflora*** **leaves**

Treatment	Dose (mg/ear)	Ear weight differences (mg)	Inhibition percentage (%)
Control (TPA + acetone)	— — —	13.5 ± 0.6	— — —
Indomethacin	0.5	7.4 ± 0.8	52.4 ± 6.2***
***Cryptostegia grandiflora***			
Total extract	1.0	7.6 ± 0.1	42.1 ± 0.9***
Ether fraction	1.0	8.1 ± 0.8	41.8 ± 8.0***
Dichloromethane fraction	1.0	6.1 ± 0.5	52.9 ± 4.3***
Methanol fraction	1.0	12.2 ± 0.4	6.8 ± 3.2

Results are expressed as mean ± S.M.E. (*n* = 6) (***p <0.001 ANOVA, statistically significant compared with control group).

**Figure 1:**
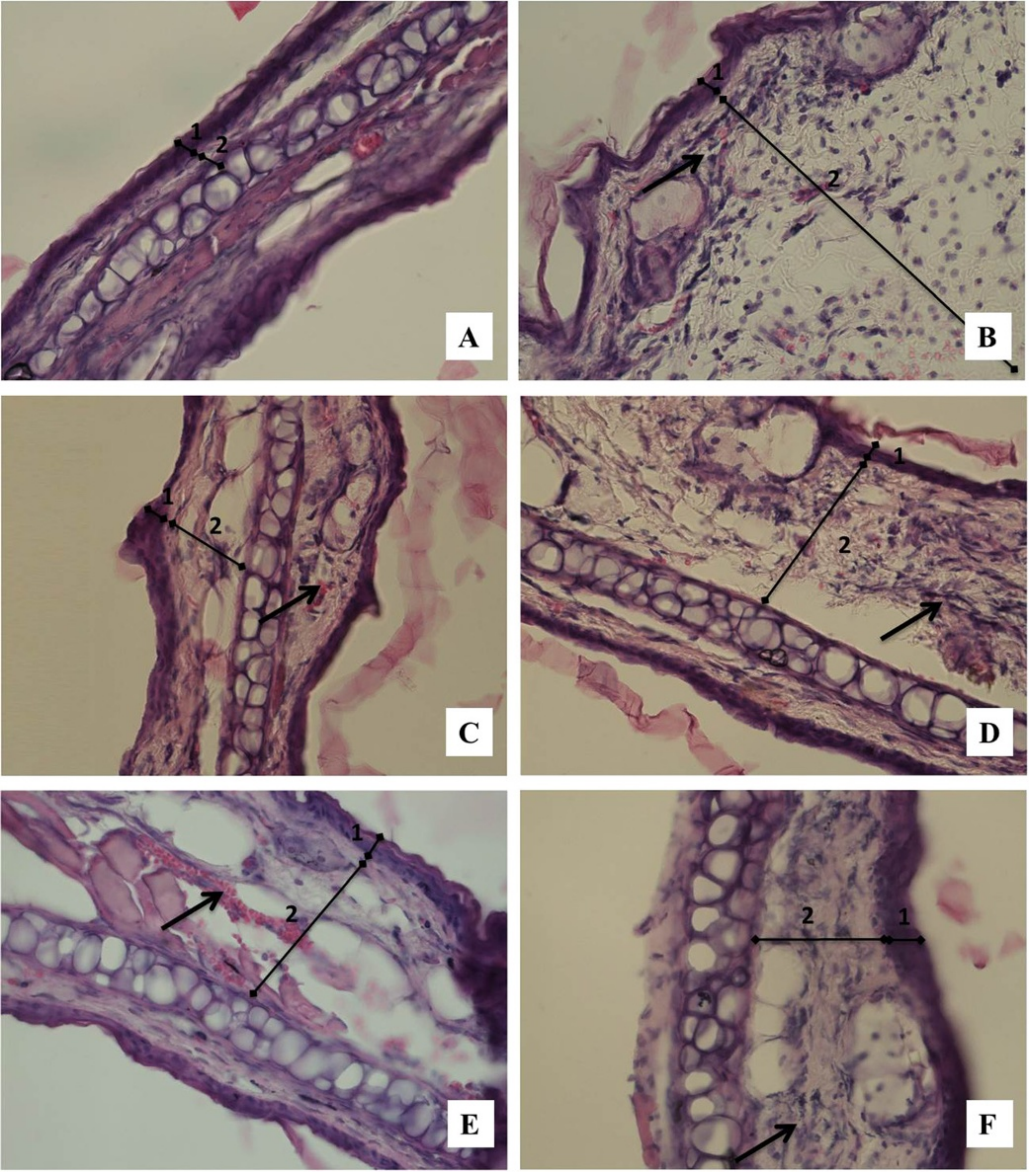
**Histopathological changes produced by total extract and primary fractions of** ***Cryptostegia grandiflora*** **, qualified by a blinded pathologist.** Pictures represents sections from mice ears stained with hematoxylin and eosin (40X) from: **(A)** Healthy group, **(B)** Control group (TPA, 2.5 μg/ear), **(C)** TPA and Indomethacin (0.5 mg/ear), **(D)** TPA and *Cryptostegia grandiflora* total extract (1.0 mg/ear), **(E)** TPA and *Cryptostegia grandiflora* ether fraction (1.0 mg/ear), and **(F)** TPA and *Cryptostegia grandiflora* dichloromethane fraction (1.0 mg/ear). The numbers 1 and 2 indicate epidermis and dermis, respectively. Arrows indicate cell infiltration into the dermis.

TPA promoted a marked increase in ear thickness, with clear evidence of edema, substantial neutrophil infiltration, and hyperplasia and hypertrophy of dermis and epidermis (Figure 1B). Treatment with *Cryptostegia grandiflora* total extract (Figure 1D) and ether (Figure 1E) and dichloromethane fractions (Figure 1F) notably reduced the total score, established by a blinded pathologist, over 45%.

### MPO activity

TPA treatment induced an increase in the tissue MPO activity. Ether and dichloromethane fractions from *Cryptostegia grandiflora* have a significant inhibitory effect on the activity of this enzyme by over 96%, showing higher activity than the positive control indomethacin (Table 2), which showed inhibition of 77.7 ± 2.2%.

**Table 2:** **Effect of total extract and primary fractions obtained from** ***Cryptostegia grandiflora*** **leaves on MPO activity in ear tissue**

Treatment	Dose (mg/ear)	MPO activity (U/mg of tissue)	Inhibition percentage (%)
Control (TPA + acetone)	— — —	18150 ± 2012.3	—
Indomethacin	0.5	4040 ± 406.9	77.7 ± 2.2***
***Cryptostegia grandiflora***			
Total extract	1.0	11040 ± 987.8	39.1 ± 5.4***
Ether fraction	1.0	600 ± 89.4	96.6 ± 0.4***
Dichloromethane fraction	1.0	666 ± 98.8	96.3 ± 0.5***

Results are expressed as mean ± S.M.E. (*n* = 6) (***p < 0.001 ANOVA, statistically significant compared with control group).

Treatment with total extract of *Cryptostegia grandiflora* leaves reduced inflammation and MPO activity in ear tissue. Such bioactivity is kept in its ether and dichloromethane fractions, which have different polarity, suggesting the presence of structurally diverse phytoconstituents that might be exerting its activity by different molecular mechanisms, contributing additively to the global anti-inflammatory effect. These results are very promissory since tested fractions constitute a mixture of many secondary metabolites, providing the first step of a bio-guided isolation conducted to isolate compounds with higher anti-inflammatory activity. Complementarily, our results suggests the usefulness of *Cryptostegia grandiflora* extracts and primary fractions to treat inflammatory skin diseases, including psoriasis and atopic dermatitis, without exerting signals of systemic toxicity. However, a detailed study of toxicity and dermal absorption will warrant its safety use as phytotherapy.

Overall, the anti-inflammatory effect of ether and dichloromethane fractions of *Cryptostegia grandiflora* is similar or higher to that observed for indomethacin, anti-inflammatory drug used as a reference [21, 22]. Therefore, these promissory fractions were selected to evaluate their effect on NO and PGE2 production by LPS-induced macrophages RAW 264.7 and their scavenging effect of DPPH, ABTS and NO radicals, in order to get further insights into the mechanisms related with the anti-inflammatory activity.

### *In vitro*effect of *Cryptostegia grandiflora*on RAW 264.7 macrophages viability

Macrophages activation with LPS induces the production of NO• and PGE2, two potent pro-inflammatory mediators [23]. NO• acts as signaling molecule that promotes growth and activity of immune cells [24]. Meanwhile, PGE2 induces arteriolar vasodilatation, increasing of vascular permeability and corporal temperature, and the pain produced by bradykinin, and serotonin [25]. In this sense, reducing the production of these inflammatory mediators is considered an alternative to prevent or treat complications associated with diseases related with inflammatory processes.

**Figure 2:**
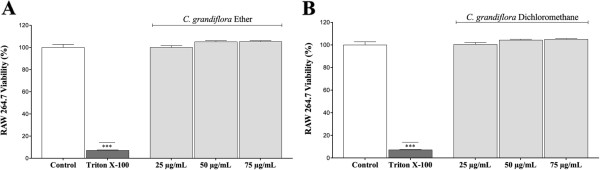
**Effect of (A) ether and (B) dichloromethane fractions obtained from** ***Cryptostegia grandiflora*** **on cell viability of RAW 264.7 macrophages.** Cytotoxicity was measured with MTT assay. Each value represents mean ± S.E.M. (*n* = 15). ***p < 0.001, compared with control group.

To guarantee that test fractions do not cause cell death, we assessed their effect on cell viability employing MTT assay. As can be seen in Figure 2, cell viability for all tested concentrations of *Cryptostegia grandiflora* fractions was similar to that obtained for control group (not treated cells), with viability percentages over 95%. In this way, concentrations below 75 μg/mL can be securely evaluated using macrophages RAW 264.7.

### Inhibitory activity of *Cryptostegia grandiflora*on NO• and PGE2 production induced by LPS

Treatment with LPS to RAW 264.7 macrophages induced synthesis and release of NO• and PGE2. Upon exposure to LPS, macrophages increased nitrite production, of up to 21.9 ± 0.7 μM, whereas cells in resting state released trace amounts of NO•. 1400 W, a selective inhibitor of inducible nitric oxide synthase (iNOS), caused a decrease in LPS-induced NO• production by more than 70% at 10 μM.

**Figure 3:**
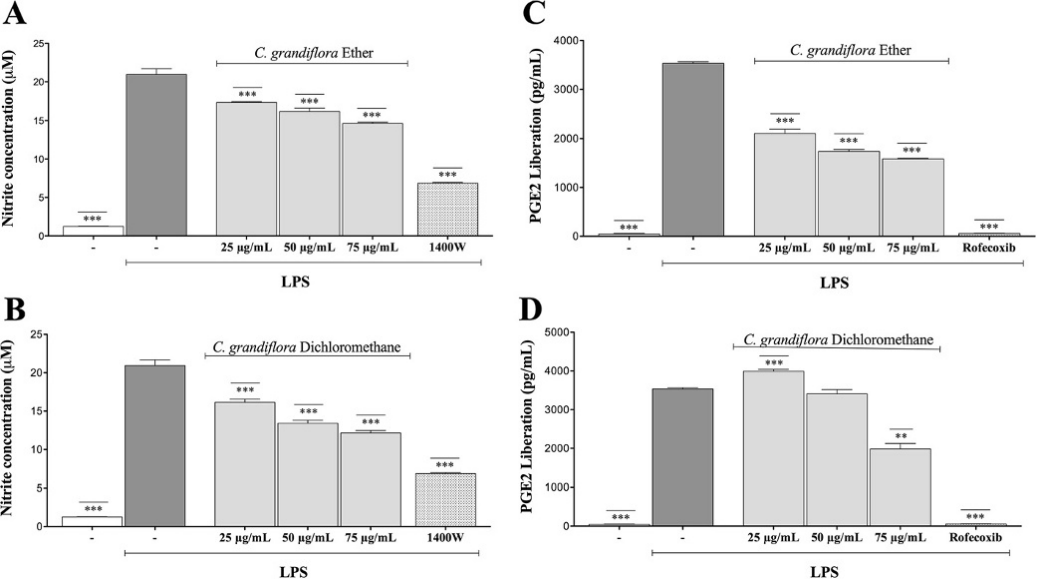
**Inhibitory effect on NO• and PGE2 production by ether (A and C) and dichloromethane (B and D) fractions from** ***Cryptostegia grandiflora*** **on RAW 264.7 cells stimulated with LPS (10 μg/mL) for 24 h.** 1400 W (10 μM) and Rofecoxib (10 μM) were employed as positive controls. Nitrite concentration in the culture medium was measured with the Griess reagent. PGE2 production was determined using a competitive ELISA kit (R&D Systems, USA). Experiments were carried out in triplicate and results show the mean ± S.E.M. (*n* = 12). **p < 0.01, ***p < 0.001, compared with LPS treated group.

Ether and dichloromethane fractions from *Cryptostegia grandiflora* diminished the production of NO• in a concentration-dependent manner, reducing significantly nitrite concentration in culture medium compared to the group treated with LPS alone (Figure 3A and 3B). On the other hand, ether and dichloromethane fractions did not show scavenging effects on NO• free radical (data not shown), therefore the reduction of nitrite concentration can be attributed directly to a blockade in NO• production in LPS-induced RAW 264.7 macrophages.

Macrophages RAW 264.7, in resting state, released 44.2 ± 11.3 pg/mL of PGE2 during 24 h of incubation. However, upon exposure to LPS alone, the macrophages increased PGE2 production, of up to 3532.3 ± 32.1 pg/mL (80 fold increase). Levels of LPS-induced PGE2 were decreased by ether and dichloromethane fractions of *Cryptostegia grandiflora* (75 μg/mL) to 1583.5 ± 15.9 and 1980.6 ± 143.4 pg/mL, respectively. In general, ether fraction was more potent than dichloromethane, diminishing levels of PGE2 in a dose dependent-manner, corresponding to a 59.6% at 75 μg/mL, 49.2% at 50 μg/mL, and 44.8% at 25 μg/mL, whereas dichloromethane fraction was only active at the highest test concentration producing 56.1% of inhibition (Figure 3C and 3D).

The above date demonstrated that ether and dichloromethane fractions from *Cryptostegia grandiflora* leaves decreased the production of NO• and PGE2. This inhibitory effect might be contributing significantly to the mechanism of the *in vivo* anti-inflammatory effect of tested fractions.

### Anti-oxidant activity

*Cryptostegia grandiflora* fractions, showed a potent scavenging effect of DPPH and ABTS free radicals, in a concentration-dependent manner, with IC_50_ values lower than 550 and 101 μg/mL, respectively (Table 3). Even though these fractions did not present comparable activity to that presented by ascorbic acid, used as reference control; they constitute a promissory source to isolate bioactive compounds with antioxidant properties, as these complex fractions are constituted by an elevated number of compounds.

**Table 3:** **Antioxidant activity of ether and dichloromethane fractions from** ***Cryptostegia grandiflora*** **leaves**

DPPH	IC_50_(μg/mL)	Confidence intervals (μg/mL)
Ascorbic acid	10.9	(10.2-11.8)
Ether fraction	549.2	(505.3-593.5)
Dichloromethane fraction	485.9	(440.9-531.0)
**ABTS**		
Ascorbic acid	2.3	(2.1-2.4)
Ether fraction	101.0	(92.7-109.2)
Dichloromethane fraction	96.9	(80.9-112.2)

Results are expressed as mean ± S.M.E. (*n* = 9).

Considering that inflammatory processes, especially those which are presented chronically, are associated with an overproduction of free radicals that induce oxidative stress and cause the onset of various degenerative diseases [9, 26], and the potent scavenging effect of DPPH and ABTS free radicals exerted by ether and dichloromethane fractions of *Cryptostegia grandiflora*, we consider that the important antioxidant activity of this plant might be a key contributing factor to the reduction of edema in animals treated with TPA. Analgesic activity of a methanolic extract of leaves of *Cryptostegia grandiflora* was recently described, attributing the significant pain reduction to the presence of flavonoids, alkaloids, tannins, saponins, terpenoids and phenolic compounds, especially to flavonoids and alkaloids analgesic principles acting against PG pathway and oxidative stress [27]. Considering that flavonoids has been described as anti-inflammatory compounds with several mechanism explaining their effect, including antioxidant and radical scavenging activities and modulation of the activities of arachidonic acid metabolism enzymes (phospholipase A2, cyclooxygenase and lipooxygenase) and nitric oxide synthase, their presence in leaves extracts of *Cryptostegia grandiflora* can be directly related to the anti-inflammatory activity demonstrated by us [28].

Conversely, the soluble protein fraction from the whole latex of *Cryptostegia grandiflora* has been described to induce a consistent inflammatory activity in different experimental models *in vivo*, regardless the administration route [12]. In this respect the latex of *Cryptostegia grandiflora* appears to be different from that of *Calotropis procera* Ait, R.Br (Apocynaceae), which produces both pro-inflammatory and anti-inflammatory effects depending on the protein content and administration route [29]. Our results, might suggest that latex contains different classes of proteins and secondary metabolites with contrasting bioactivities, providing a plentiful field for new research.

In conclusion, further work is needed in order to identify the specific antioxidant and anti-inflammatory compounds, as well as to clarify the potential synergic effects that might help to explain the mechanism of *Cryptostegia grandiflora* extract and fractions.

## Conclusions

Our study demonstrated the anti-inflammatory activity of the extract and primary fractions obtained from the leaves of *Cryptostegia grandiflora*, which appears to be the first report establishing this biological activity and supporting the folk use of this plant in the Colombian Caribbean region. The promissory results obtained in this work constitutes the basis for further studies aiming to isolate, purify and characterize the bioactive anti-inflammatory compounds from *Cryptostegia grandiflora* and get new insights into the related molecular mechanisms.

## Methods

### Reagents

12-*O*-tetradecanoyl-phorbol-13-acetate (TPA), indomethacin, 2,2-diphenyl-1-picrylhydrazyl (DPPH), ascorbic acid, 2,2’-azinobis-(3-ethylbenzothiazoline-6-sulfonic acid) (ABTS), potassium persulfate (K_2_S_2_O_8_), Dulbecco’s Modified Eagle Medium (DMEM), L-glutamine, antibiotics (Penicillin-Streptomycin), dimethyl sulfoxide (DMSO), trypan blue, lipopolysaccharides from *Escherichia coli* serotype 0127:B8 (LPS), (N-[[3-(aminomethyl)phenyl]methyl]-ethanimidamide dihydrochloride (1400 W), rofecoxib, sodium nitrite (NaNO_2_), sodium nitroprusside (SNP), N-(1,1-naphthyl)ethylenediamine dihydrochloride, sulfanilamide, Phosphate Buffer Saline (PBS) tablets, *O*-Dianisidine, ethylenediaminetetraacetic acid (EDTA), hematoxylin and eosin were purchased from Sigma-Aldrich (St. Louis, MO, USA). fetal bovine serum (FBS) was obtained from GIBCO (Gaithersburg, MD, USA). Hydrogen peroxide (H_2_O_2_), hexadecyl-trimethylammonium bromide (HETAB), and 3-(4,5-dimethyl-thiazol-2-yl)-2,5-diphenyl-tertazolium bromide (MTT) from Calbiochem® (San Diego, CA, USA). Organic solvents were analytical grade and obtained from Mallinckrodt Baker (San Diego, CA, USA).

### Plant material

Leaves of *Cryptostegia grandiflora* (Apocynaceae) were collected at Pueblo Nuevo, Bolivar, Colombia (4° 10° 44’ 33” N; 75° 15’ 28” W; elevation 3 m.a.s.l.), in March 2011. The plant material was identified by Felipe A. Cardona N., and the voucher specimen has been deposited with the identification number HUA-175330 at the Herbarium University of Antioquia, Medellin, Colombia.

### Preparation of ethanol extract and primary fractions

Dried and powdered leaves (689.9 g) were exhaustively extracted with ethanol by maceration at room temperature (25 ± 3°C). The extract was then filtered and concentrated in a rotary evaporator under controlled temperature (35–45°C) and reduced pressure. A portion of the concentrated total extract (10 g) was fractioned using liquid/liquid partition procedures with ether (1.4 g), dichloromethane (1.5 g), and methanol (3.8 g).

### Experimental animals

Female ICR mice weighing 20–25 g were provided by Instituto Nacional de Salud, Colombia. Animals were allowed to acclimatize for 10 days before use and fed with standard rodent food and water *ad libitum*. They were housed in filtered-capped polycarbonate cages and kept in a controlled environment with temperature at 22 ± 3°C and relative humidity between 65 to 75%, under a cycle of 12 h light/darkness. All the procedures were performed in accordance with ethical guidelines on the care and use of animals in laboratory research, which were approved by the Ethics Committee of the University of Cartagena.

### TPA-induced acute ear edema

The edema was induced by topical application of TPA (2.5 μg/ear) in acetone, according to the method described by De Young *et al*. [30]. Mice in groups (*n* = 6) were treated on the inner and outer surfaces of the right ear with an acetone solution containing test extracts/fractions from *Cryptostegia grandiflora* leaves (1 mg/ear in 20 μL of vehicle) or Indomethacin (0.5 mg/ear in 20 μL of vehicle), as reference drug. At the same time, the TPA solution was applied to both faces of the right ear. The left ear received only acetone, as a control. Four hours after the inflammation induction, animals were sacrificed by cervical dislocation and a disk (6 mm diameter) was removed from both ears (treated and untreated) and individually weighed on an Ohaus Pioneer™ (PA214) analytical balance. The degree of edema was indicated by the increase in the weight of the right ear disk over the left. The anti-inflammatory activity was expressed as percentage of the inhibition of edema in treated groups in comparison to control group (vehicle) [10].

Histopathologic analysis was performed according to standard protocols [31]. Ear samples were preserved in buffered formalin, embedded in paraffin, cut into 5 μm sections, and stained with hematoxylin eosin. Subsequently, ear tissue was analyzed by a blinded pathologist using light microscopy, which assessed the presence of edema, epidermal hyperplasia/hypertrophy, hyperkeratosis, infiltration of mononuclear and polymorphonuclear cells, connective tissue disruption, and dermal fibrosis, using a scale of 0 to 4 (0, none; 1, mild; 2, mild; 3; moderate; 4, severe).

### Myeloperoxidase (MPO) assay

MPO activity was measured according to the method by [32]. Ear tissue was weighed and homogenized in 10 vol of 50 mM PBS, pH 7.4. The homogenate was centrifuged at 4000 rpm for 30 min at 4°C. The pellet was again homogenized in 10 vol of 50 mM PBS, pH 6.0, containing 0.5% HETAB. The homogenate was subjected to cycles of freezing/thawing and brief periods of sonication (15 s) and centrifugation at 4000 rpm for 10 min at 4°C. Supernatants (20 μL) were mixed with 50 μL of *o*-dianisidine dihydrochloride (0.067%) and 50 μL of hydrogen peroxide (0.003%) in each well of a 96-well microplate, and incubated for 5 min. Enzyme activity was determined by measuring changes in the Optical Density at 450 nm (OD_450_), using a microplate reader (Multiscan EX Thermo®), and expressed as the inhibition percentage of MPO levels calculated against the absorbance presented by the control group.

### Cell culture

The murine RAW 264.7 macrophage-like cell line was obtained from the American Type Culture Collection (TIB-71; Rockville, MD, USA) and routinely cultured in DMEM supplemented with 2 mM L-glutamine, antibiotics (100 IU/mL of penicillin-100 μg/mL streptomycin) and heat-inactivated FBS at 37°C in a humidified atmosphere containing 5% CO_2_.

### MTT assay

The mitochondrial-dependent reduction of MTT to formazan was used to assess the possible cytotoxic effects of *Cryptostegia grandiflora* leaves on RAW 264.7 cells [33, 34]. Macrophages (2×10^5^ cells/mL) were cultured at 37°C and allowed to grow to confluence. Subsequently, the culture medium was replaced with various concentrations of test extracts/fractions or 1400 W for 30 min, followed by stimulation with of *E. coli* LPS (1 μg/mL). Control group was incubated with the same amount of DMSO. Triton X-100 (2%) was used as positive control. After 24 h, the medium was removed and cells incubated with MTT solution (3 mg/mL). Four hours later medium was carefully aspirated, formazan crystals were dissolved in DMSO, and OD_550_ was measured using a microplate reader (Multiscan EX Thermo®).

### NO• and PGE2 production

The evaluation of the anti-inflammatory activity was performed in a manner similar to that described for cell viability. In brief, RAW 264.7 cells (2×10^5^ cells/mL) were allowed to grow to confluence at 37°C. The adherent cells were treated for 30 min with various concentrations of fractions from *Cryptostegia grandiflora* leaves, 1400 W or Rofecoxib, and stimulated with LPS (1 μg/mL). Control cells were cultured under the same conditions but were not exposed to the effect of LPS. Twenty four hours later, culture supernatants were collected and stored at −20°C until use.

NO• release was determined spectrophotometrically by the accumulation of nitrite (NO_2_^−^), using the Griess reaction [35]. Briefly, 100 μL of cell culture medium was mixed with 100 μL of Griess reagent (1:1 mixture of 0.1% N-(1-naphthyl) ethylenediamine dihydrochloride and 1% sulfanilamide in 5% H_3_PO_4_), and incubated at room temperature for 5 min. The OD_550_ of the samples was measured using a microplate reader (Multiscan EX Thermo®) and compared with a standard curve prepared with NaNO_2_ (1–200 μM). Levels of PGE2 were determined using commercially available competitive ELISA kits (R&D Systems, Minneapolis, MN) according to the manufacturer’s instructions. Final results were expressed as pg/mL of supernatant.

### NO radical scavenging effect

NO-scavenging effect of fractions of *Cryptostegia grandiflora* leaves was determined by the method of [36] with some modfications. Sodium nitroprusside (SNP) in aqueous solution spontaneously generates NO• which interacts with oxygen to produce NO_2_^−^ ions that can be estimated using Griess reagent. Scavengers of NO• compete with oxygen, leading to reduced production of NO_2_^−^ [37]. Briefly, SNP (5 mM) in PBS, pH 7.4, was mixed with test compounds and incubated at 25°C for 120 min. After this period, samples were incubated with an equal volume of Griess reagent for 5 min. The OD_550_ was measured using a microplate reader (Multiscan EX Thermo®) and compared with standard solutions of NaNO_2_ (1–200 μM).

### DPPH radical scavenging activity

DPPH-scavenging effect was determined using the method described by [38], with some modifications. In brief, 75 μL of fractions from *Cryptostegia grandiflora* and ascorbic acid, at various concentrations, were plated into 96-well plates, mixed with 150 μL of DPPH (100 μg/mL; in methanol), and incubated at room temperature (25 ± 3°C). Vehicles were used as negative controls. After 30 min, the disappearance of DPPH radical absorption was determined at OD_550_ using a microplate reader (Multiscan EX Thermo®).

### ABTS radical scavenging activity

ABTS-scavenging effect was determined using the method described by [39], whit modifications. Briefly, 10 μL of various concentrations of fractions from *Cryptostegia grandiflora* or ascorbic acid, used as positive controls, were mixed with 190 μL of ABTS (3.5 mM; in ethanol) and K_2_S_2_O_8_ (1.25 mM). The mixture was incubated at room temperature for 5 min. After this time, the disappearance of ABTS radical absorption was determined at OD_405_ using a microplate reader (Multiscan EX Thermo®).

### Statistical analysis

Results from three independent assays were expressed as mean ± standard error of the mean (S.E.M) and analyzed using one-way analysis of variance (ANOVA), followed by Dunnett’s or Tukey’s *post hoc* test, to determine the differences between groups. Values of p < 0.05 were considered significant. LC_50_ and IC_50_ values were calculated using non-linear regression analysis and expressed as the mean and its 95% confidence interval.
